# Strain-driven band inversion and topological aspects in Antimonene

**DOI:** 10.1038/srep16108

**Published:** 2015-11-05

**Authors:** Mingwen Zhao, Xiaoming Zhang, Linyang Li

**Affiliations:** 1 School of Physics and State Key Laboratory of Crystal Materials, Shandong University, Jinan 250100, Shandong, China

## Abstract

Searching for the two-dimensional (2D) topological insulators (TIs) with large bulk band gaps is the key to achieve room-temperature quantum spin Hall effect (QSHE). Using first-principles calculations, we demonstrated that the recently-proposed antimonene [Zhang *et al.*, *Angew. Chem. Int. Ed.* 54, 3112–3115 (2015)] can be tuned to a 2D TI by reducing the buckling height of the lattice which can be realized under tensile strain. The strain-driven band inversion in the vicinity of the Fermi level is responsible for the quantum phase transition. The buckled configuration of antimonene enables it to endure large tensile strain up to 18% and the resulted bulk band gap can be as large as 270 meV. The tunable bulk band gap makes antimonene a promising candidate material for achieving quantum spin Hall effect (QSH) at high temperatures which meets the requirement of future electronic devices with low power consumption.

Motivated by the successful discovery and application of graphene, there has been extensive research into other two-dimensional (2D) materials^1^,^2^,^3^. Some of them were demonstrated to be superior to graphene in specific aspects. For example, graphene was firstly proposed as a 2D topological insulator with a bulk band gap due to spin-orbit coupling (SOC) and gapless edge states protected by time-reversal symmetry^4^,^5^,^6^,^7^. The edge states characterized by Dirac-cone-like linear energy dispersion are quite promising for the realization of conducting channels without dissipation due to the robustness against backscattering^8^,^9^. However, the SOC effect in graphene is very week, leading to an unobservably small bulk band gap (~10^−3^ meV). The critical temperature to achieve dissipationless transport in graphene is unrealistically low^10^,^11^,^12^,^13^. This drawback can be overcome in other 2D materials of group IV elements, such as silicene^14^, germanene^14^, stanene^15^, silicon carbide^16^, stanane^17^, carbon nitrides^18^,^19^,^20^, and those of group III–V compounds, such as GaAs^21^ and GaBi^22^. The SOC in these materials is much stronger than that in graphene and the bulk band gaps are consequently larger. For example, upon chloridization, the nontrivial bulk band gap in GaBi monolayer can be as large as 0.95 eV^22^. Therefore, the critical temperature for achieving dissipationless transport in this material will be above room temperature. This significant improvement is related to the electron band inversion and buckled configurations of these 2D materials.

The 2D materials of group V elements are gaining considerable interest because of the unique properties. Phosphorene, a 2D material that can be isolated through mechanical exfoliation from layered black phosphorus, is a normal semiconductor with a sizable band gap (1.2–2.2 eV). A field effect transistor (FET) action has been demonstrated in a few layer phosphorene by manipulating the doping level via back-gate voltage, leading to on-off ratio of the order of 10^5^ 23. A recent theoretical work showed that few-layer phosphorene can be tuned to a 2D topological insulator with a bulk band gap of about 5 meV by applying perpendicular electric field^24^. The small bulk band gap is due to the weak SOC of light elements. For bismuth which is the heaviest group V element, SOC is naturally strong, and Bi(111) bilayer was predicted to be a 2D TI with a large bulk band gap^25^. This 2D bismuth materials have been successfully grown on Bi_2_Te_3_ substrates^26^ or locally exfoliated from bulk crystal^27^, and the existence of the edge states in Bi(111) bilayer was found^28^,^29^. Very recently, antimonene, a new 2D material of group V element, was proposed on the basis of first-principles calculations^30^. The isolation of antimonene was expected to be achieved by exfoliating layered Sb crystal, thanks to the weak interlayer interaction. In contrast to the semimetallic bulk Sb crystal, antimonene becomes semiconducting when it is chinned to one atomic layer. However, unlike Bi(111) bilayer, antimonene proposed in that work is a normal semiconductor with a band gap of 2.28 eV^30^. Questions naturally arise: whether antimonene can be tuned to a TI and how robust the topological nontriviality will be?

Using first-principles calculations within density-functional theory (DFT), we demonstrated that this goal can be reached by reducing the buckling height of the lattice under biaxial tensile strain larger than 14.5%. The transition from a normal semiconductor to a nontrivial topological insulator was attributed to the strain-driven band inversion in the vicinity of the Г point. The reducing buckling height modifies the hybridization of atomic orbitals, leading to band inversion and enhancement of SOC. The bulk band gap due to SOC increases with the increase of tensile strain. The buckled configuration of antimonene enables it to endure large tensile strain up to 18% and the bulk band gap can be as large as 270 meV. Compared with the chemical-fuctionalization strategy, e.g. halogenated 2D materials, such strain-induced phase transition is reversible and controllable. This interesting result implies that antimonene is a promising candidate material for achieving quantum QSHE at high temperatures which meets the requirement of future electronic devices with low power consumption.

## Results

**Figure 1:**
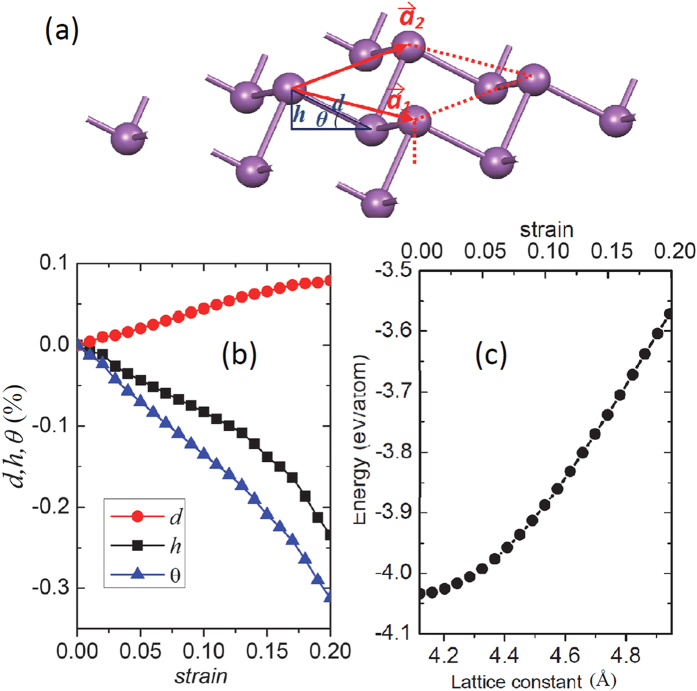
(**a**) Schematic representation of the buckled configuration of antimonene. (**b**) structural evolution represented by three parameters indicated in (**a**) of antimonene under tensile strain. (**c**) Variation of energy in response to tensile strain.

Antimonene has the point group symmetry of *D*_*3d*_ with spatial inversion included, as shown in Fig. 1(a). Similar to the case of Bi(111) bilayer, it exhibits a bipartite honeycomb lattice with A and B sublattices. The two sublattices have different heights, forming a buckled configuration. At the equilibrium state, the Sb-Sb bond length is about 2.89 Å and the height of buckling (*h*) is 1.64 Å with an angle of θ = 26.8°, as indicated in Fig. 1(a). The length of the basis vectors is 4.12 Å, slightly longer than that reported in ref. 30. The formation energy calculated from the energy difference between antimonene and isolated Sb atoms is about −4.034 eV/atom.

Buckled configuration was always expected to sustain larger tensile strain than planner one. We therefore studied the structural and energetic evolution under biaxial tensile strain. In our calculations, biaxial tensile strain was applied by fixing the lattice constant to a series of values longer than that of the equilibrium state and optimizing the atomic coordinates. The variation of Sb-Sb distance (*d*), buckling height (*h*) and buckling angle (*θ*) as a function of tensile strain (τ) is plotted in Fig. 1(b). The formation energy of antimonene at each tensile strain is shown in Fig. 1(c). With the increase of tensile strain, the Sb-Sb distance increases gradually, while both buckling height and buckling angle decrease rapidly. At τ = 0.18, the Sb-Sb bond is only stretched by 7.9%, but *h* and *θ* are reduced by 18.6% and 27.6%, respectively. The Sb-Sb covalent bonds of antimonene are preserved in this tensile strain range. This leads to slight energy increase, as shown in Fig. 1(c). The energy increase at τ = 0.18 is about 0.396 eV/atom, only 9.8% of the value of the equilibrium state.

**Figure 2:**
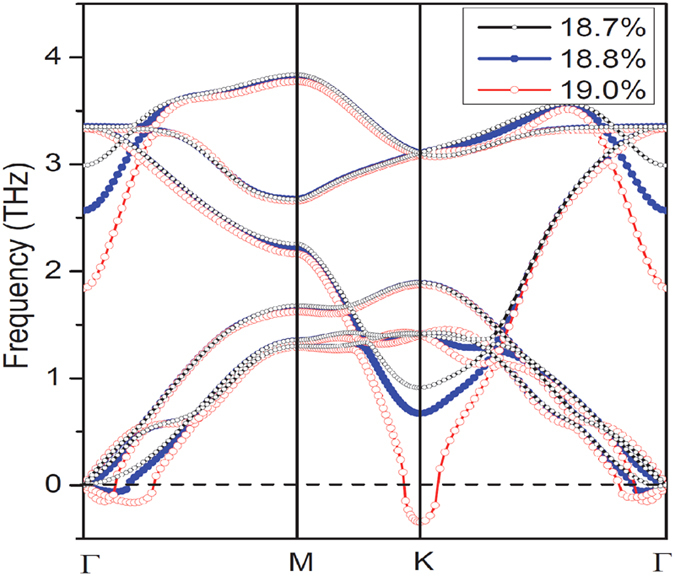
Phonon spectra of antimonene under the biaxial tensile strains of 18.7%, 18.8%, and 19%, respectively, obtained from DFT. The negative frequencies correspond to the imaginary frequency modes which are dynamically unstable.

We also checked the dynamic stability of antimonene under tensile strain. When the tensile stain is lower than 18.7%, no imaginary frequency modes were found, implying that the stretched antimonene is dynamically stable. However, when the tensile stain exceeds the critical value, the imaginary frequency modes come to appear, as shown in Fig. 2, indicating that the antimonene becomes dynamically unstable. Therefore, in the following parts, we focus on the electronic properties of the antimonene under the tensile stain lower than the critical value.

**Figure 3:**
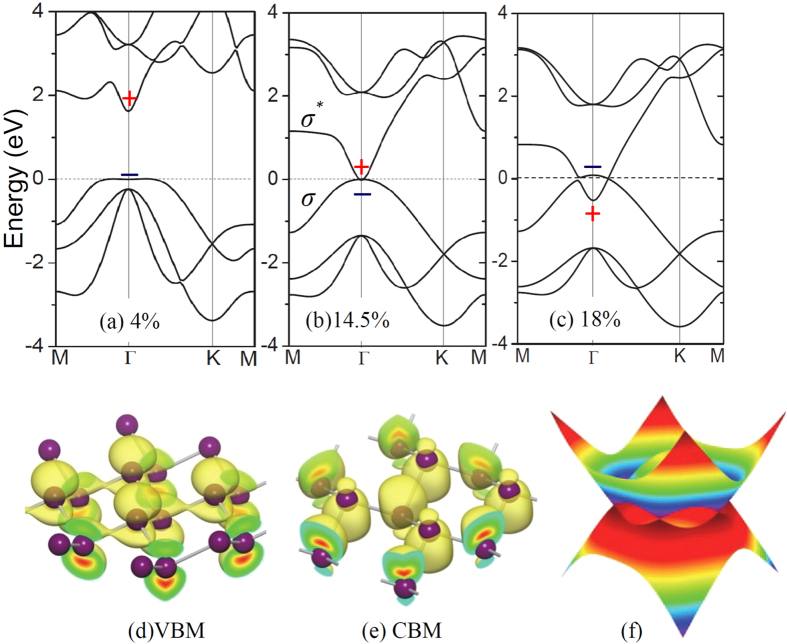
(**a–c**) Electronic band structures of antimonene under different tensile strains obtained from DFT calculation within HSE functional. The energy at the Fermi level was set to zero. (**d,e**) Electron density profiles of the wavefuctions of the valence band maximum (VBM) and the conduction band minimum (CBM) of the antimonene under tensile strain of 4%. (**f**) Two-dimensional electron band structure of the antimonene under tensile strain of 18% in the vicinity of the Dirac points. The signs (+/-) in (**c**) indicate the parities of the wavefunctions at the Г point.

The electronic structure modification of antimonene in response to tensile strain is shown in Fig. 3. At the tensile strain of τ = 0.04, antimonene converts to a direct-band-gap semiconductor with a band gap of 1.98 eV at the Г point. The density profiles of the electron wavefunctions (WFs) at the valence band maximum (VBM) and the conduction band minimum (CBM) can be featured as σ and σ* states of *p*_*z*_ atomic orbitals of Sb, as indicated by the isosurfaces of the WFs shown in Fig. 3(d,e), respectively. The band gap decreases with the increase of tensile strain and comes to close at τ = 0.145, as shown in Fig. 3(b). When the tensile strain is further increased, σ-σ* band inversion takes place in the region near the Г point, as shown Fig. 3(c). A small band gap along the Г-M direction is opened up due to the σ-σ* coupling, while the meeting point between valence and conduction bands is preserved along the Г-K direction, giving rise to six Dirac cones with a six-fold symmetry in Brillouin zone, as shown in Fig. 3(f). It is noteworthy that the Dirac cone in this stretched antimonene is titled with strong directional anisotropy in the reciprocal space, which would lead to interesting properties that differ significantly from the Dirac-Fermions in graphene.

To illustrate the band inversion mechanism explicitly, we proposed a tight-binding (TB) model of *s, p*_*x,*_ *p*_*y*_ and *p*_*z*_ atomic orbitals. The effective Hamiltonian was taken as:


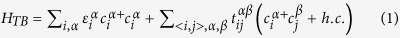


Here, 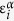, 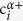, and 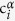 represent the on-site energy, creation, and annihilation operators of an electron at the α-orbital of the *i*-th atom. The 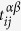 parameter is the nearest-neighbor hopping energy of an electron between an α-orbital of *i*-th atom and β-orbital of *j*-th atom, 
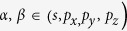
. According to TB theory, the hopping energies can be evaluated on the basis of four parameters (*V*_*ssσ*_, *V*_*spσ*_, *V*_*ppσ*_, and *V*_*ppπ*_) in combined with the atomic coordinates of antimonene (see Supporting Materials). For simplification, the values of *V*_*ssσ*_, *V*_*spσ*_, *V*_*ppσ*_, and *V*_*ppπ*_ were supposed to be independent of the tensile strain. This is reasonable because the Sb-Sb bond length changes slightly, especially at small tensile strain. We started from the semiconducting antimonene with a direct band gap which can be reproduced by the TB Hamiltonian. For the stretched antimonene, the buckling angle was changed to the data of DFT calculations to represent the structure deformation in response to tensile strain. Using this simple TB Hamiltonian, we found that the band gap decreases with the decrease of buckling angle and comes to close at θ = 27.7°, corresponding to the tensile strain of 14.5%, which is in good agreement with our DFT results. As the buckling angle is further reduced, band inversion takes place in the vicinity of the Γ point. Interestingly, the band gap opened along the Γ-M direction due to the σ-σ* coupling can also be reproduced using this TB model. This implies that the strain-induced band inversion is mainly due to buckling angle reducing under tensile strain, rather than the changes of Sb-Sb bond. We also varied the *V*_*ssσ*_, *V*_*spσ*_, *V*_*ppσ*_, and *V*_*ppπ*_ to reflect the elongation of the Sb-Sb bond, but found that the evolution of the band lines is insensitive to the changes of these parameters. The strain-driven band inversion in antimonene differs significantly from the band inversion in Bi bilayer which is caused by SOC^31^.

**Figure 4:**
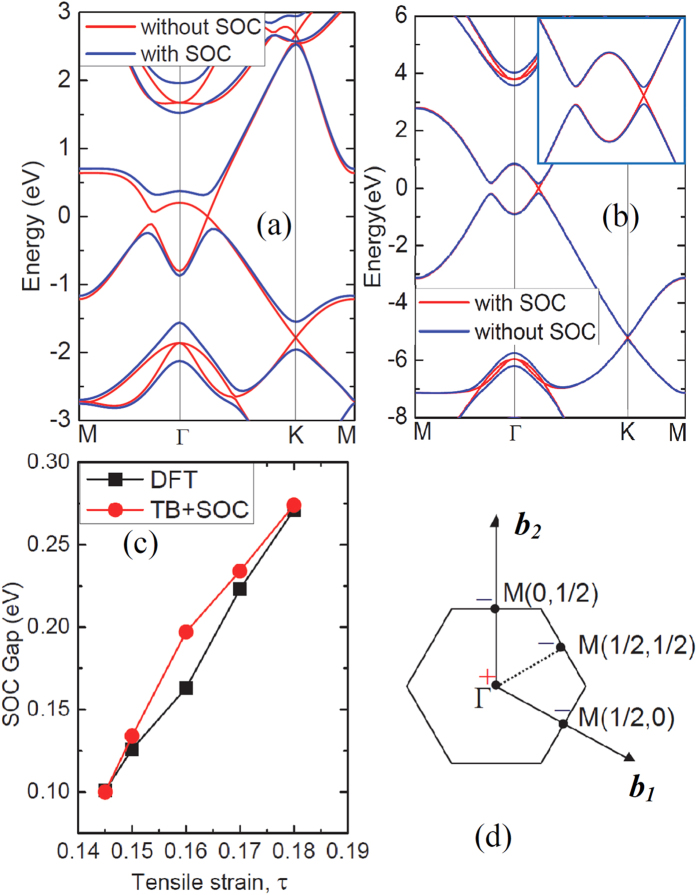
(**a,b**) Electronic band structures of the antimonene under the tensile strain of 18% obtained from (**a**) DFT calculations within HSE functional and (**b**) tight-binding model with (blue lines) and without (red lines) spin-orbit coupling (SOC). (**c**) Evolution of band gaps opened due to SOC in response to tensile strain. (**d**) The parities of the occupied bands at the time-reversal invariant momentum.

We then turned on SOC in our DFT calculations of electronic band structures. Unsurprisingly, a band gap is opened at the Dirac point of antimonene, as shown in Fig. 4(a). The SOC gap increases with the increase of tensile strain and can be as large as 270 meV at the tensile strain of τ = 0.18, as shown in Fig. 4(c). The variation trend of the SOC gap can be well reproduced by introducing a spin-orbit term into the TB Hamiltonian (see Fig. 4(b,c) and the Supporting Information).

The topological nontriviality of stretched antimonene can be confirmed by a nonzero topological invariant Z_2_. Due to the inversion symmetry of antimonene, the Z_2_ index can be deduced from the knowledge of the parities of the four time-reversal and parity invariant points at Brillouin zone, without having to know about the global properties of the energy bands, as proposed by Fu *et al.*^32^ The honeycomb lattice of antimonene has four time-reversal invariant momenta at the point of 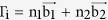, with 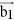 and 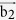 being the basis vectors of the reciprocal lattice and n_1_, n_2_ϵ{0, 1/2}. The Z_2_ invariant ν is defined by


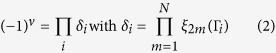


for 2N occupied bands. 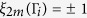 is the parity eigenvalue of the *2m*-th occupied energy band at the time-reversal invariant momentum 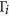. Our first-principles calculations showed as the tensile strain is lower than the critical value 14.5%, ***δ***_***i***_has the values of (-), (-), (-), and (-) at (0, 0), (1/2, 0), (0, 1/2), and (1/2, 1/2) time-reversal momenta, respectively, leading to zero Z_2_ invariant. In this case, antimonene is a trivial insulator. When the tensile strain exceeds the critical value, however, the ***δ*** value at the Γ point changes to (+) due to the band inversion near the Γ point, as shown in Fig. 3(c). The topological invariant is therefore Z_2_ = 1, indicating that the stretched antimonene becomes a topological insulator.

**Figure 5:**
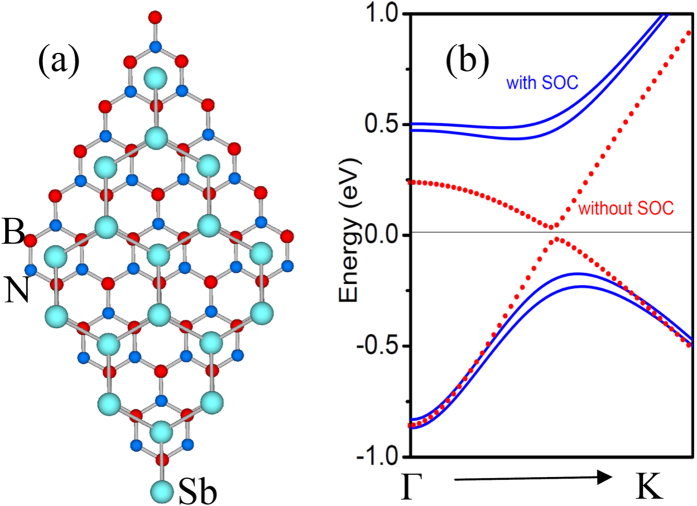
(**a**) The superstructure of antimonene grown on a BN substrate. (**b**) Electronic band structures of the Sb/BN superstructure in the vicinity of Fermi level (set to zero) with and without SOC.

Finally, we checked whether the topological properties in the stretched antimonene can remain on substrates or not. We placed stretched antimonene (with τ = 0.18) on top of a 2 × 2 hexagonal BN substrate which was expected to have a weak van der Waals interfacial interaction and right hexagonal symmetry, as shown in Fig. 5(a). The optimized interlayer spacing between antimonene and the BN substrate is about 3.33 Å. Our first-principles calculations show that the QSH states remain intact. The SOC gaps opened at the Dirac points are intact, as shown in Fig. 5(b). These results demonstrate the feasibility of attaining the QAH states of the antimonene grown on a substrate.

## Discussion

The mechanism of the strain-induced QSH states in antimonene differs significantly from that reported for other 2D materials, such as GaAs^21^, where it was attributed to the weakening of the covalent bonds. For antimonene, our TB model clearly showed that the band inversion is due to the changes of the bond angles, i.e. the buckling angles denoted in Fig. 1(a), rather than the bond elongation. The changes of buckling angles modify the hybridization of the atomic orbitals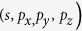 and consequently the alignment of the bands, giving rise to band inversion. Controllability and reversibility are the advantages of this mechanism. Additionally, the critical tensile strain of 14.5% that triggers the quantum phase transition is basically unrealistic for bulk Sb materials, but accessible for ultrathin films. For example, the uniaxial and biaxial strains larger than 10% have been achieved experimentally in graphene which has a planar configuration^33^,^34^. Our simulations have demonstrated that antimonene can sustain high tensile strain (~18%), thanks to the buckling configuration. Therefore, similar strategies may be useful for the experimental realization of high tensile strains in antimonene.

In summary, based on the first-principles calculations combined with a tight-binding model, we demonstrated that antimonene can be tuned to a topological insulator by applying biaxial tensile strain larger than 14.5%. The quantum phase transition from a normal semiconductor to a TI is closely related to the strain-induced σ-σ* band inversion in the vicinity of the Γ point. The SOC gap increases with the increase of tensile strain. The buckled configuration of antimonene enables it to sustain large tensile strain up to 18%, which gives rise to a SOC gap of 270 meV. These interesting results make antimonene a promising candidate material for achieving quantum spin Hall effect (QSH) at high temperatures which meets the requirement of future electronic devices with low power consumption.

## Methods

All the calculations were performed using the plane wave basis Vienna ab initio simulation package known as VASP code^35^,^36^. The ion-electron interactions were described using projector-augmented-wave potentials^37^. A generalized gradient approximation (GGA) in the form proposed by Perdew, Burke, and Ernzerhof (PBE)^38^ was employed to treat the electron exchange-correlation functional. The atomic positions were relaxed until the maximum force on each atom was less than 0.01 eV/Å. The energy cutoff of the plane waves was set to 600 eV with the energy precision of 10^−8^ eV. The Brillouin zone was sampled by using an 11 × 11 × 1 Gamma-centered Monkhorst-Pack grid. Antimonene was modeled by unit cells repeated periodically on the x-y plane, while a vacuum region of about 15 Å was applied along the z-direction to exclude the interactions between images. In the electronic structure calculations, Heyd-Scuseria-Ernzerhof (HSE) screened Coulomb hybrid density functional^39^ was adopted to correct the shortcoming of the PBE functional which always underestimated the band gaps and consequently the critical tensile strain of band inversion^40^. SOC was included by a second variational procedure on a fully self-consistent basis. The phonon spectra were calculated on the basis of the force constants obtained from a supercell approach within the PHONON code in combination with VASP.

## Additional Information

**How to cite this article**: Zhao, M. *et al.* Strain-driven band inversion and topological aspects in Antimonene. *Sci. Rep.* **5**, 16108; doi: 10.1038/srep16108 (2015).

## Supplementary Material

Supporting Information

## References

[b1] Tang Q., Zhou Z. & Chen Z. F. Innovation and discovery of graphene-like materials via density-functional theory computations, Wires Comput. Mol. Sci. 5, 360–379 (2015).

[b2] Wang Z. H. *et al.* Phagraphene: A low-energy graphene allotrope composed of 5-6-7 carbon rings with distorted Dirac cones, Nano Lett. 15, 6182–6186 (2015). 26262429 10.1021/acs.nanolett.5b02512

[b3] He T. *et al.* Layered titanium oxide nanosheet and ultrathin nanotubes: a first-principles prediction, J. Phys. Chem. C 113, 13610–13615 (2009).

[b4] Kane C. L. & Mele E. J. Quantum spin Hall effect in graphene. Phys. Rev. Lett. 95, 226801 (2005). 16384250 10.1103/PhysRevLett.95.226801

[b5] Hasan M. Z. & Kane C. L. Colloquium: Topological insulators. Rev. Mod. Phys. 82, 3045–3067 (2010).

[b6] Qi X.-L. & Zhang S.-C. Topological insulators and superconductors. Rev. Mod. Phys. 83, 1057–1110 (2011).

[b7] Xiao-Liang Q. & Shou-Cheng Z. The quantum spin Hall effect and topological insulators. Phys. Today 63, 33–38 (2010).

[b8] Bernevig B. A., Hughes T. L. & Zhang S.-C. Quantum spin Hall effect and topological phase transition in HgTe quantum wells. Science 314, 1757–1761 (2006). 17170299 10.1126/science.1133734

[b9] König M. *et al.* Quantum spin Hall insulator state in HgTe quantum wells. Science 318, 766–770 (2007). 17885096 10.1126/science.1148047

[b10] Yao Y., Ye F., Qi X.-L., Zhang S.-C. & Fang Z. Spin-orbit gap of graphene: First-principles calculations. Phys. Rev. B 75, 041401 (2007).

[b11] Min H. *et al.* Intrinsic and Rashba spin-orbit interactions in graphene sheets. Phys. Rev. B 74, 165310 (2006).

[b12] Boettger J. C. & Trickey S. B. First-principles calculation of the spin-orbit splitting in graphene. Phys. Rev. B 75, 121402 (2007).

[b13] Gmitra M., Konschuh S., Ertler C., Ambrosch-Draxl C. & Fabian J. Band-structure topologies of graphene: Spin-orbit coupling effects from first principles. Phys. Rev. B 80, 235431 (2009).

[b14] Liu C.-C., Feng W. & Yao Y. Quantum spin Hall effect in silicene and two-dimensional germanium. Phys. Rev. Lett. 107, 076802 (2011). 21902414 10.1103/PhysRevLett.107.076802

[b15] Xu Y. *et al.* Large-gap quantum spin Hall insulators in tin films. Phys. Rev. Lett. 111, 136804 (2013). 24116803 10.1103/PhysRevLett.111.136804

[b16] Zhao M. & Zhang R. Two-dimensional topological insulators with binary honeycomb lattices: SiC_3_ siligraphene and its analogs. Phys. Rev. B 89, 195427 (2014).

[b17] Chen X., Li L. & Zhao M. Dumbbell stanane: a large-gap quantum spin hall insulator, Phys. Chem. Chem. Phys. 17, 16624–16629 (2015). 26037067 10.1039/c5cp00046g

[b18] Wang A., Zhang X. & Zhao M. Topological insulator states in a honeycomb lattice of s-triazines, Nanoscale 6, 11157–11162 (2014). 25119110 10.1039/c4nr02707h

[b19] Zhang X., Wang A. & Zhao M. Spin-gapless semiconducting graphitic carbon nitrides: A theoretical design from first principles. Carbon 84, 1–8 (2015).

[b20] Zhang X. & Zhao M. Prediction of quantum anomalous Hall effect on graphene nanomesh, Rsc Adv. 5, 9875–9880 (2015).

[b21] Zhao M., Chen X., Li L. & Zhang X. Driving a GaAs film to a large-gap topological insulator by tensile strain. Sci. Rep. 5, 8441 (2015). 25676173 10.1038/srep08441 PMC5389133

[b22] Li L., Zhang X., Chen X. & Zhao M. Giant topological nontrivial band gaps in chloridized gallium bismuthide. Nano Lett. 15, 1296–1301 (2015). 25625786 10.1021/nl504493d

[b23] Li L. *et al.* Black phosphorus field-effect transistors. Nat Nanotechnol. 9, 372–377 (2014). 24584274 10.1038/nnano.2014.35

[b24] Liu Q., Zhang X., Abdalla L. B., Fazzio A. & Zunger A. Switching a normal insulator into a topological insulator via electric field with application to phosphorene. Nano Lett. 15, 1222–1228 (2015). 25607525 10.1021/nl5043769

[b25] Murakami S. Quantum spin Hall effect and enhanced magnetic response by spin-orbit coupling. Phys. Rev. Lett. 97, 236805 (2006). 17280226 10.1103/PhysRevLett.97.236805

[b26] Hirahara T. *et al.* Interfacing 2D and 3D Topological Insulators: Bi(111) Bilayer on Bi_2_Te_3_. Phys. Rev. Lett. 107, 166801 (2011). 22107414 10.1103/PhysRevLett.107.166801

[b27] Sabater C. *et al.* Topologically protected quantum transport in locally exfoliated bismuth at room temperature. Phys. Rev. Lett. 110, 176802 (2013). 23679755 10.1103/PhysRevLett.110.176802

[b28] Yang F. *et al.* Spatial and energy distribution of topological edge states in single Bi(111) bilayer. Phys. Rev. Lett. 109, 016801 (2012). 23031123 10.1103/PhysRevLett.109.016801

[b29] Drozdov I. K. *et al.* One-dimensional topological edge states of bismuth bilayers. Nat. Phys. 10, 664–669 (2014).

[b30] Zhang S., Yan Z., Li Y., Chen Z. & Zeng H. Atomically thin arsenene and antimonene: semimetal-semiconductor and indirect-direct band-gap transitions. Angew. Chem. Int. Ed. 54, 3112–3115 (2015). 10.1002/anie.201411246 25564773

[b31] Zhou M. *et al.* Epitaxial growth of large-gap quantum spin Hall insulator on semiconductor surface. Proc. Natl. Acad. Sci. USA 111, 14378–14381, (2014). 25246584 10.1073/pnas.1409701111 PMC4210051

[b32] Fu L. & Kane C. L. Topological insulators with inversion symmetry. Phys. Rev. B 76, 045302 (2007).

[b33] Pérez Garza H. H., Kievit E. W., Schneider G. F. & Staufer U. Controlled, reversible, and nondestructive generation of uniaxial extreme strains (>10%) in graphene. Nano Lett. 14, 4107–4113 (2014). 24872014 10.1021/nl5016848

[b34] Shioya H., Craciun M. F., Russo S., Yamamoto M. & Tarucha S. Straining graphene using thin film shrinkage methods. Nano Lett. 14, 1158–1163 (2014). 24490629 10.1021/nl403679f PMC3962252

[b35] Kresse G. & Hafner J. Ab initio molecular dynamics for open-shell transition metals. Phys. Rev. B 48, 13115–13118 (1993). 10.1103/physrevb.48.13115 10007687

[b36] Kresse G. & Furthmuller J. Efficient iterative schemes for ab initio total-energy calculations using a plane-wave basis set. Phys. Rev. B 54, 11169–11186 (1996). 10.1103/physrevb.54.11169 9984901

[b37] Kresse G. & Joubert D. From ultrasoft pseudopotentials to the projector augmented-wave method. Phys. Rev. B 59, 1758–1775 (1999).

[b38] Perdew J. P., Burke K. & Ernzerhof M. Generalized gradient approximation made simple. Phys. Rev. Lett. 77, 3865–3868 (1996). 10062328 10.1103/PhysRevLett.77.3865

[b39] Heyd J., Scuseria G. E. & Ernzerhof M. Hybrid functionals based on a screened Coulomb potential. J Chem. Phys. 118, 8207–8215 (2003).

[b40] Chuang F. C., Hsu C. H., Chen C. Y., Huang Z. Q., Ozolins V. O., Lin H. & Bansil A. Tunable topological electronic structure in Sb(111) bilayers: A first-principles study, Appl. Phys. Lett. 102, 022424 (2013).

