# Supplementary material for: Strain-driven band inversion and topological aspects in Antimonene
Source: Sci Rep. 2015 Nov 5;5:16108. doi: 10.1038/srep16108 (PMC4633587; doi:10.1038/srep16108)
Supplement: Supporting Information [file srep16108-s1.pdf]

Supporting Information for

Strain-driven band inversion and topological aspects in Antimonene

Mingwen Zhao, Xiaoming Zhang, Linyang Li

School of Physics and State Key Laboratory of Crystal Materials, Shandong University, Jinan 250100, Shandong, China

(E-mail: zmw@sdu.edu.cn)

Tight-binding mode (TB) for antimonene under tensile strain

The effective Hamiltonian is taken as:

$$H_{TB} = \sum_{i,\alpha} \varepsilon_i^{\alpha} c_i^{\alpha+} c_i^{\alpha} + \sum_{<i,j>,\alpha,\beta} t_{ij}^{\alpha\beta} (c_i^{\alpha+} c_j^{\beta} + h.c.)$$

Here, ε_i^{α} , $c_i^{\alpha+}$, and c_i^{α} represent the on-site energy, creation, and annihilation operators of an electron at the α-orbital of the *i*-th atom. The $t_{ij}^{\alpha\beta}$ parameter is the nearest-neighbor hopping energy of an electron between an α-orbital of *i*-th atom and β-orbital of *j*-th atom, $\alpha, \beta \in (s, p_x, p_y, p_z)$. According to TB theory, the hopping energies can be evaluated on the basis of four parameters ($V_{ss\sigma}$, $V_{sp\sigma}$, $V_{pp\sigma}$, and $V_{pp\pi}$) in combined with the atomic coordinates of the antimonene..

$$t_{ij}^{ss} = V_{ss\sigma}$$

$$t_{ij}^{sp_x} = V_{sp\sigma} \times cos\theta$$

$$t_{ij}^{sp_y} = V_{sp\sigma} \times cos\varphi$$

$$t_{ij}^{p_xp_x} = V_{pp\sigma} \times cos^2\theta + V_{pp\pi} \times sin^2\theta$$

$$t_{ij}^{p_yp_y} = V_{pp\sigma} \times cos^2\varphi + V_{pp\pi} \times sin^2\varphi$$

$$t_{ij}^{p_xp_y} = (V_{pp\sigma} - V_{pp\pi}) \times cos\,\theta \times sin\,\varphi$$

θ and φ are the angles of the vector pointed from *i*-th atom to *j*-th atom with respect to x- and y-axis. The on-site energies of *s*- and *p*-orbital are set to ε^s= -21.85 eV and ε^p = -8.30 eV. Other parameters optimized at the equilibrium state are $V_{ss\sigma}$ = -2.79 eV, $V_{sp\sigma}$ = 2.718 eV, $V_{pp\sigma}$ = 5.832 eV, and $V_{pp\pi}$ = -1.548 eV, respectively.

We started from the semiconducting antimonene with a direct band gap which can be reproduced by the TB Hamiltonian. When a tensile strain is applied to the antimonene, the buckling angle was changed to the data of DFT calculations to describe the structure deformation in response to the tensile strain. Using this simple TB Hamiltonian, we found that the band gap decreases with the decrease of buckling angle and comes to close at θ = 27.7°, corresponding to a tensile strain of 14.5%, which is in good agreement with DFT results. As the buckling angle is further reduced, band inversion takes place in the vicinity of the Γ point. Interestingly, the band gap opened along the Γ-M direction due to the σ-σ* coupling can also be reproduced using this TB model. This implies that the strain-induced band inversion is mainly due to buckling angle reducing under tensile strain, rather than the changes of Sb-Sb bond.

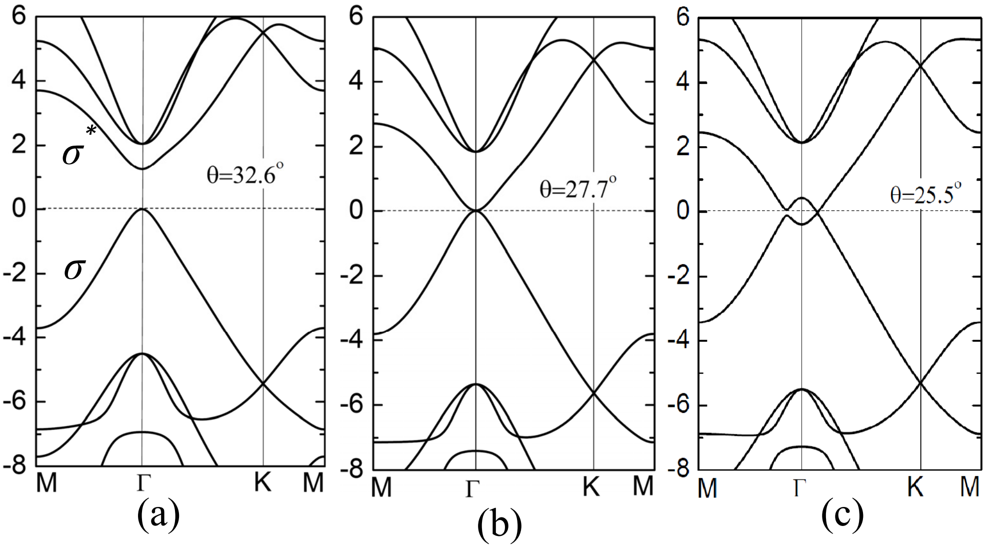

FIG. S1 Tight-binding electronic band lines in the vicinity of the Fermi level (set to zero) of the antimonene under different tensile strains represented by different buckling angles.
